# Improvement of LV Reverse Remodeling Using Dynamic Programming of Fusion-Optimized Atrioventricular Intervals in Cardiac Resynchronization Therapy

**DOI:** 10.3389/fcvm.2021.700424

**Published:** 2021-08-20

**Authors:** Zhongkai Wang, Pan Li, Bili Zhang, Jingjuan Huang, Shaoping Chen, Zhuhong Cai, Yingyi Qin, Jihai Fan, Wendong Tang, Yongwen Qin, Ruogu Li, Xianxian Zhao

**Affiliations:** ^1^Department of Cardiology, Changhai Hospital, Second Military Medical University, Shanghai, China; ^2^Department of Cardiology, Shanghai Chest hospital, Shanghai Jiao Tong University, Shanghai, China; ^3^Department of Ultrasound, Changhai Hospital, Second Military Medical University, Shanghai, China; ^4^Department of Health Statistics, Second Military Medical University, Shanghai, China; ^5^Department of Cardiology, 455th Hospital of Nanjing Military Command, Shanghai, China

**Keywords:** atrioventricular delay, optimization, heart failure, echocardiography, cardiac resynchronization therapy

## Abstract

**Background:** The patient-tailored SyncAV algorithm shortens the QRS duration (QRSd) beyond what conventional biventricular (BiV) pacing can. However, evidence of the ability of SyncAV to improve the cardiac resynchronization therapy (CRT) response is lacking. The aim of this study was to evaluate the impact of CRT enhanced by SyncAV on echocardiographic and clinical responses.

**Methods and Results:** Consecutive heart failure (HF) patients from three centers treated with a quadripolar CRT system (Abbott) were enrolled. The total of 122 patients were divided into BiV+SyncAV (*n* = 68) and BiV groups (*n* = 54) according to whether they underwent CRT with or without SyncAV. Electrocardiographic, echocardiographic, and clinical data were assessed at baseline and during follow-up. Echocardiographic response to CRT was defined as a ≥15% decrease in left ventricular end-systolic volume (LVESV), and clinical response was defined as a NYHA class reduction of ≥1. At the 6-month follow-up, the baseline QRSd and LVESV decreased more significantly in the BiV+SyncAV than in the BiV group (QRSd −36.25 ± 16.33 vs. −22.72 ± 18.75 ms, *P* < 0.001; LVESV −54.19 ± 38.87 vs. −25.37 ± 36.48 ml, *P* < 0.001). Compared to the BiV group, more patients in the BiV+SyncAV group were classified as echocardiographic (82.35 vs. 64.81%; *P* = 0.036) and clinical responders (83.82 vs. 66.67%; *P* = 0.033). During follow-up, no deaths due to HF deterioration or severe procedure related complications occurred.

**Conclusion:** Compared to BiV pacing, BiV combined with SyncAV leads to a more significant reduction in QRSd and improves LV remodeling and long-term outcomes in HF patients treated with CRT.

## Introduction

Cardiac resynchronization therapy (CRT) is an effective therapeutic modality for patients with advanced heart failure (HF), cardiomyopathy, and left bundle branch block (LBBB). It has been demonstrated that CRT improves left ventricular remodeling and clinical symptoms, and thereby reduces hospitalization and all-cause mortality (1, 2). However, these effects vary among individuals, with approximately one-third failing to respond to CRT (3). Optimizing the atrioventricular delay (AVD) guided by ultrasound or electrocardiogram (ECG) enhances the fusion of intrinsic conduction and biventricular (BiV) pacing, resulting in improved CRT responses and clinical outcomes (4–6). Post-programmed AVD, however, fails to adapt to the dynamics of each patient due to heart rate variability and/or drugs (7, 8).

SyncAV is a novel, device-based algorithm that can dynamically adjust AVD to synchronize the BiV pacing and intrinsic conduction (i.e., a triple wavefront fusion) (6, 9) to fit each patient's changing needs. Previous studies have reported that SyncAV narrows the QRS duration (QRSd) by optimizing pacemaker parameters (10–12). However, these studies have been limited to QRSd measurements, so the impact of SyncAV on cardiac function remains unclear. This study compared the echocardiographic and clinical improvements in CRT patients equipped with SyncAV vs. those lacking the SyncAV function.

## Methods

### Patient Selection

In total, 122 consecutive patients who underwent successful CRT implantations from 2017 to 2019 at Changhai Hospital, Shanghai Chest Hospital, and the 455th Hospital of Nanjing Military Command were retrospectively studied. The inclusion criteria were as follows: patients with New York Heart Association (NYHA) functional Class II to IV HF despite optimal medical therapy; left ventricular ejection fraction (LVEF) ≤35%, sinus rhythm, and a QRSd ≥130 ms with or without LBBB. According to Strauss criteria, LBBB was defined as (1) QRSd ≥140 ms for males or ≥130 ms for females, (2) mid-QRS notch or slur in ≥2 leads: I, aVL, V_1_, V_2_, V_5_, V_6_, and (3) rS or QS in V_1_/V_2_ (with R peak time <60 ms) (13). Patients with life expectancy ≤1 year, intrinsic PR intervals ≥300 ms, RBBB (right bundle branch block), degree II or III AV block, severe aortic valve stenosis or regurgitation, new-onset MI within 4 months, or persistent atrial arrhythmia (including atrial tachycardia, atrial flutter, and atrial fibrillation [AF]) were excluded.

Of the 122 patients treated with CRT pacemakers ([CRT-P], Quadra Allure 3,140 or Quadra Allure 3,242; Abbott), 68 patients with CRT-P devices equipped with SyncAV function were divided into the BiV+SyncAV group, while 54 patients receiving CRT-P devices without SyncAV function were included in the BiV group. The selection of the type of pacemaker implantation (CRTP with or without SyncAV function) was at the discretion of the treating physician. Basic demographics, HF data, relevant anti-HF medications, QRSd, LV lead locations, electrocardiographic recordings, and echocardiographic parameters were collected. Informed consent was obtained from all included patients. The protocol was approved by the Ethics Committee of Changhai Hospital, Shanghai Chest Hospital, and 455th Hospital of Nanjing Military Command. Studies were performed in accordance with the principles established in the Declaration of Helsinki. Patients or the public were not involved in the design, or conduct, or reporting, or dissemination plans of our research.

### Study Protocol

CRT was performed according to clinical practice recommendations. Briefly, CRT devices were implanted *via* the cephalic, axillary, or subclavian vein under local anesthesia. Right ventricle (RV) and right atrium (RA) leads were placed in the RV apex or low septum and RA appendage, respectively. CRT patients were implanted with a quadripolar LV lead (Abbott, Quartet™ 1458Q/St. Jude Medical) termed D1, M2, M3, and P4, from distal to proximal. The site of the last LV lead was selected based on coronary sinus venography, lead stability, and the pacing threshold, preferably in the lateral or posterolateral vein.

### Electrocardiographic Assessments

**Figure 1:**
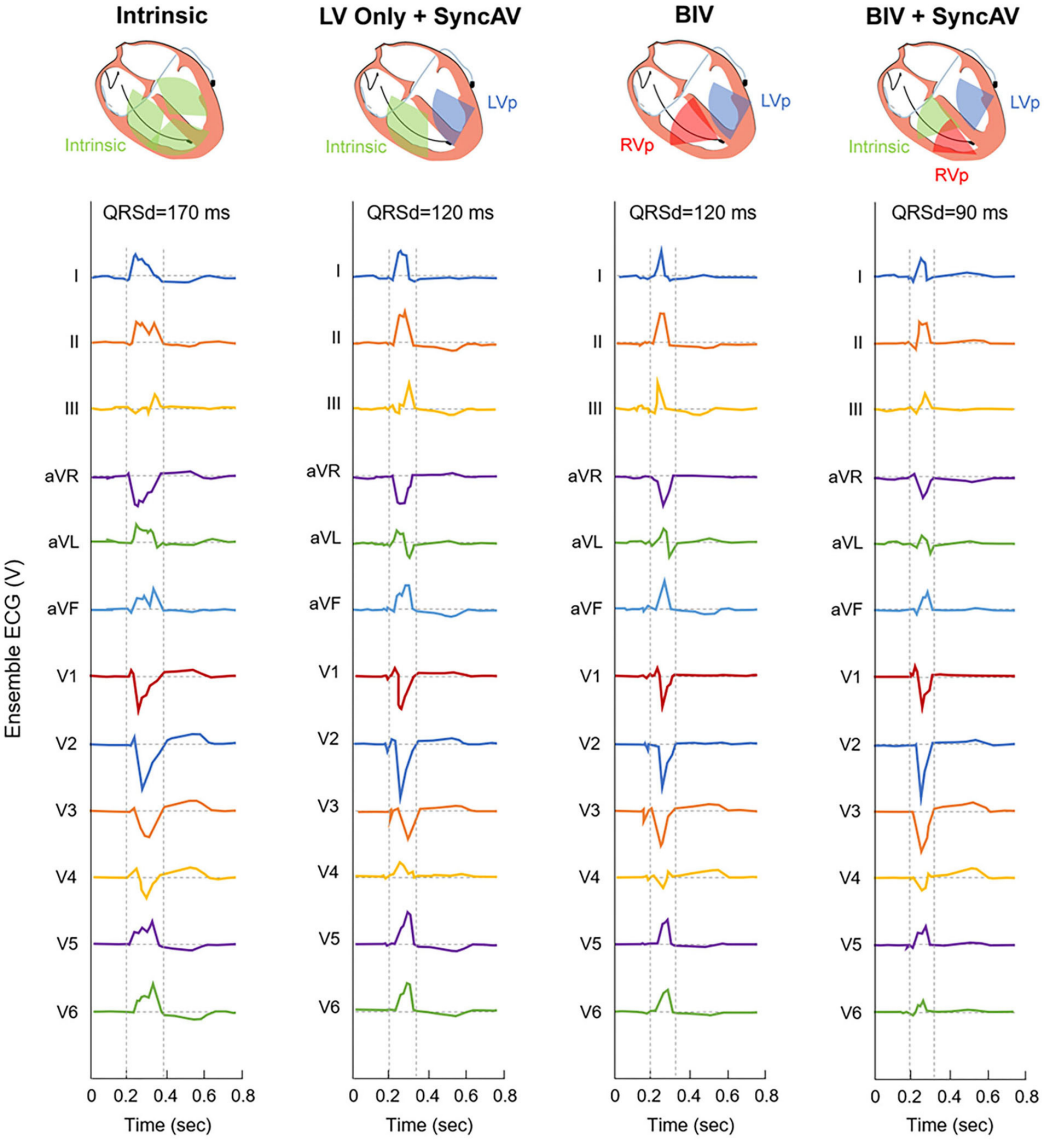
Representative 12-lead ECG for QRS interval at intrinsic and three different pacing modes during post-implant optimization in a single patient.

The PR interval, QRSd, and QRS morphology were recorded prior to CRT implantation. During BiV pacing using individual electrodes, QRSd, the time from the onset of the QRS complex in lead II to the first larger positive or negative peak in the LV EGM (QLV) (14), QLV normalized to the intrinsic QRSd (QLV/QRSd), and the interval of the first large positive or negative peaks of RV and LV electrograms (RV-LV interval) were measured using intracardiac electrograms (IEGM) and 12-lead ECGs at a speed of 100 mm/s (15, 16). In the BiV+SyncAV group, devices were temporarily programmed to Mode I: LV-only+SyncAV (offset: 50 ms); Mode II: BiV with nominal settings (paced/sensed AVD of 140/110 ms) or Mode III: BiV+SyncAV (optimal offset: 10–100 ms). QRSd measurements were performed at three pacing modes (Figure 1). Paced QRSd was measured from the beginning of the rapid deflection to the end of the QRS complex (17). QRSd was analyzed by an ECG technician who was blinded to the settings. Each sample was analyzed for 10 min and repeated three times. Averages were sequentially calculated.

### Echocardiographic Assessments

Echocardiograms were assessed using a Vivid E95 ultrasound system (General Electric Healthcare, Chalfont St Giles, UK). Echocardiographic parameters, including LVEF, left ventricular end-diastolic volume (LVEDV), LV end-systolic volume (LVESV), LV end-systolic dimension (LVESD), and LV end-diastolic dimension (LVEDD) were assessed prior to implantation and 6 months postoperatively. The aortic velocity time integral (aVTI) was obtained from the apical five-chamber view using a pulsed-wave Doppler (PWD) sample placed on an LV outflow tract below the aortic valve according to the American Society of Echocardiography guidelines (18). As above, the aVTI values in three temporarily programmed modes of patients in the BiV+SyncAV group were also measured. CRT settings were programmed at least 15 min prior to assessments. Measurements were the average of three consecutive cardiac cycles.

### CRT Device Optimization

CRT devices were optimized 48 h post-implantation using the programmer (Abbott Inc., Sylmar, CA, USA). The first step was aimed at selecting the best LV pacing site and vector. The capture threshold for the four LV pacing sites was assessed in VDD mode (fixed paced AV delay of 120 ms). The SyncAV CRT offset was sequentially reprogrammed from 10 to 100 ms, based on the pacing site to yield the narrowest QRSd, and this was considered the optimal offset. The VV interval (LV-RV) was fixed at 30 ms in our study to ensure the LV pacing 30 ms ahead of RV pacing. For patients in the BiV group, the nominal AV (paced/sensed AVD of 140/110 ms) and VV (LV-RV 30 ms) delay were used to program the CRT.

### Follow-Up Protocol

Clinical symptoms, 6-min walk distances (6MWT), Minnesota Living with Heart Failure (MLHF) score, NYHA class, the threshold of pacing, electrode impedance, electrocardiographic data, echocardiographic data, clinical medication, and adverse events were evaluated in all patients at baseline and at 6 months postoperatively. Adverse cardiovascular events and infection, electrode dislocation, thrombosis, and early pacemaker battery depletion were recorded. The main adverse cardiovascular events (MACE) included cardiac death, non-fatal MI, non-fatal stroke, and the mortality rate. The primary outcome was echocardiographic response at 6 months. The secondary end points included NYHA class, 6MWT, and MLHF Questionnaire. Clinical responses were defined as a NYHA class decrease ≥1. Echocardiographic response to CRT was defined as a ≥15% reduction in LVESV after 6 months of CRT. Additionally, non-responders were defined as those with LVESV reduction <15%; superresponders had LVESV reduction ≥30% relative to baseline (19).

### Statistical Analysis

Continuous variables are shown as the mean ± standard deviation (SD) or median with range as appropriate. Discrete variables are shown as frequencies and/or percentages. Continuous variables were compared using an unpaired Student *t*-test or Mann-Whitney Test. Chi-square or Fisher exact tests were used to compare categorical variables. Multiple comparison among the pacing modes was done using LSD-test or Kruskal-Wallis one-way ANOVA. Multivariable regression models were used to explore the outcomes between BiV+SyncAV and BiV groups when adjusted for clinical covariates, QLV/QRS. Because it is a retrospective study, we also conducted analyses with two propensity score weighting methods to reduce the effects of confounders: inverse probability weighting (IPTW) and overlap weighting (OW) (20, 21). The individual propensity score of BiV+SyncAV was estimated *via* a multivariable logistic regression model, including all characteristics. We included the absolute standardized mean differences to evaluate the balance of covariates between the two groups, and the criteria for covariate unbalance was set to 0.1. *P*-values <0.05 indicated statistical significances. All statistical analyses were performed using SPSS version 22.0.

## Results

### Baseline Characteristics

Baseline characteristics were comparable between groups (Table 1 and Supplementary Table 4). Patients received anti-HF therapy for ≥3 months prior to CRT implantation. At baseline, 58 (85.3%) patients in the BiV+SyncAV group were classified as NYHA III to IV compared to 46 (85.2%) in the BiV group (*P* = 0.987). LBBB occurred in 52 (76.5%) patients in the BiV+SyncAV group, compared to 41 (75.9%) in the BiV group (*P* = 0.944). The baseline QRSd in the BiV+SyncAV group was compared to BiV group (159.5 vs. 165.0 ms; *P* = 0.453). The median baseline PR-interval did not differ between BiV+SyncAV and BiV groups (178.0 vs. 175.0 ms; *P* = 0.738).

**Table 1:** Baseline characteristics of study population.

**Characteristics**	**Overall (*n =* 122)**	**BiV+SyncAV (*n =* 68)**	**BiV (*n =* 54)**	***P*-value**
Age (years)	66.0 (60.0, 71.00)	67.5 (58.5, 72.0)	64.0 (60.0, 69.0)	0.291
Male gender (*n*, %)	51 (41.8)	27 (39.7)	24 (44.4)	0.598
ICM (*n*, %)	26 (21.3)	14 (20.6)	12 (22.2)	0.827
BNP (pg/ml)	603.4 (312.1, 1317.4)	565.4 (339.4, 1167.9)	652.3 (291.9, 1405.0)	0.942
LBBB (*n*, %)	93 (76.2)	52 (76.5)	41 (75.9)	0.944
PR interval (ms)	176.0 (164.0, 190.0)	178.0 (160.0, 193.5)	175.0 (165.0, 188.0)	0.738
QRS duration (ms)	160.0 (153.0, 171.0)	159.5 (153.5, 170.0)	165.0 (152.0, 174.0)	0.453
LVEF (%)	34.0 (30.0, 37.0)	35.0 (30.0, 38.5)	33.0 (31.0, 35.0)	0.547
LVEDD (cm)	6.5 (5.9, 7.0)	6.4 (5.9, 6.9)	6.7 (5.8, 7.1)	0.883
LVESD (cm)	5.4 (4.9, 5.9)	5.3 (4.9, 5.8)	5.5 (4.7, 5.9)	0.747
LVEDV (ml)	212.5 (173.0, 255.0)	209.0 (173.0, 247.0)	227.5 (167.0, 264.0)	0.869
LVESV (ml)	142.0 (113.0, 173.0)	135.0 (113.0, 163.5)	148.0 (102.0, 173.0)	0.818
LA volume (ml)	79.2 (62.0, 99.0)	77.3 (63.0, 98.0)	82.9 (61.0, 100.0)	0.885
MR (mm^2^)	30.0 (14.0, 46.0)	30.0 (16.0, 44.0)	24.0 (14.0, 48.0)	0.977
Hypertension (*n*, %)	54 (44.3)	32 (47.1)	22 (40.7)	0.485
DM (*n*, %)	32 (26.2)	18 (26.5)	14 (25.9)	0.946
ACEI/ARB/ARNI (*n*, %)	107 (87.7)	60 (88.2)	47 (87.0)	0.841
β-block (*n*, %)	109 (89.3)	60 (88.2)	49 (90.7)	0.656
Diuretics (*n*, %)	103 (84.4)	57 (83.8)	46 (85.2)	0.837
MLHF score	74.0 (69.0, 79.0)	73.0 (69.0, 79.0)	75.5 (72.0, 78.0)	0.431
6MWT (m)	240.0 (180.0, 260.0)	240.0 (190.0, 263.5)	233.0 (180.0, 252.0)	0.726
NYHA class (*n*, %)
II	18 (14.8)	10 (14.7)	8 (14.8)	0.979
III	85 (69.7)	47 (69.1)	38 (70.4)	
IV	19 (15.5)	11 (16.2)	8 (14.8)	

*Values are mean ± SD or n (%) or median (interquartile range). DM, diabetes mellitus; ICM, ischemic cardiomyopathy; LBBB, left bundle branch block; MR, mitral regurgitation; LA, left atrium; MLHF, Minnesota Living with Heart Failure; 6MWT, 6-min walk distances; LVEF, left ventricular ejection fraction; LVEDD, left ventricular end-diastolic dimension; LVESD, left ventricular end-systolic dimension; LVEDV, left ventricular end-diastolic volume; LVESV, left ventricular end systolic volume*.

### Procedural Outcomes

The CRT device was successfully implanted into all patients. The final locations of the LV leads were lateral (47.1% in BiV+SyncAV vs. 48.1% in BiV), posterolateral (19.1 vs. 20.4%), anterolateral (17.6 vs. 16.7%), and posterior (11.8 vs. 9.2%) (Supplementary Figure 1). The LV lead locations were similar between groups (All *P* > 0.05) (Supplementary Table 1). All patients had pacing sites with suitable thresholds, sensing, and impedances. The mean QLV/QRS in the BiV+SyncAV group was 0.70 ± 10.6 (57.4% > 0.7) compared to 0.70 ± 8.9 (53.7% > 0.7) (*P* = 0.974) in the BiV group. The median RV-LV interval did not differ between BiV+SyncAV and BiV groups (76.0 vs. 79.0 ms; *P* = 0.509) (Supplementary Table 1).

### Electrocardiographic Outcomes

**Figure 2:**
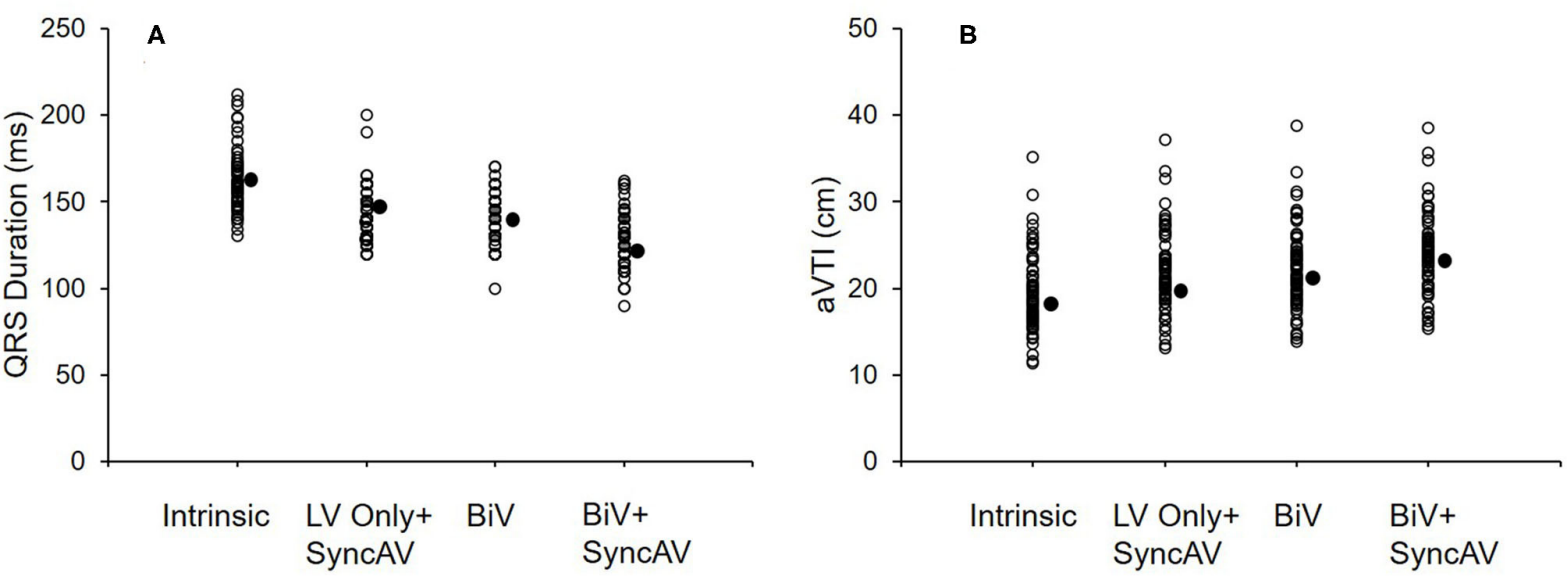
QRS interval at baseline and three different pacing modes (LV only+SyncAV, BiV, or BiV+SyncAV) in the BiV+SyncAV group **(A)**; aVTI score at baseline and three different pacing modes (LV only+SyncAV, BiV, or BiV+SyncAV) in the BiV+SyncAV group **(B)**.

Figure 2A shows the QRSd at each CRT setting relative to the intrinsic conduction in the BiV+SyncAV group. BiV performed better than LV only+SyncAV (50 ms) (138.43 ± 13.69 vs. 143.75 ± 15.19 ms; *P* < 0.05). The most significant narrowing of the QRSd complex (126.40 ± 14.89 ms; *P* < 0.001) occurred during BiV pacing coupled to SyncAV-programmed titration offsets.

At the 6-month follow-ups, a significant reduction in QRSd was found in both the BiV+SyncAV group and the BiV group (all *P* < 0.001, Table 2). Additionally, the QRSd reduction of patients with QLV/QRS >0.7 was similar to that of patients with QLV/QRS ≤0.7 in the BiV+SyncAV group (−38.23 ± 17.47 vs. −33.59 ± 14.52 ms, *P* = 0.344, Supplementary Table 2). Differences in electrocardiographic outcomes between the BiV+SyncAV group and BiV group are shown in Table 2. BiV+SyncAV led to more significant reductions in QRSd than BiV alone at 6 months (−11.68 ms, 95% CI −16.90 ~ −6.46 ms; *P* < 0.001).

**Table 2:** Improvement in QRS, clinical, and echocardiographic outcomes between groups.

**Outcomes**	**BiV+SyncAV (** ***n*** **=** **68)**		**BiV (** ***n*** **=** **54)**		**Multivariable analyses** ^***†***^	
	Delta^*^	***P*-value^**#**^**	Delta^*^	***P*-value^**#**^**	**Effect size (95% CI)^**‡**^**	***P*-value^**##**^**
Delta QRS, ms	−36.25 ± 16.33	<0.001	−22.72 ± 18.75	<0.001	−11.68 (−16.90, −6.46)	<0.001
Delta LVEF, %	13.60 ± 8.93	<0.001	6.27 ± 9.88	<0.001	7.82 (5.53, 10.11)	<0.001
Delta LVESV, ml	−54.19 ± 38.87	<0.001	−25.37 ± 36.48	<0.001	−28.86 (−38.09, −19.64)	<0.001
Delta LVEDV, ml	−60.67 ± 45.63	<0.001	−24.68 ± 42.04	<0.001	−36.99 (−48.79, −25.19)	<0.001
Delta 6MWT, m	96.84 ± 72.19	<0.001	30.61 ± 34.63	<0.001	67.18 (49.93, 84.44)	<0.001
Delta MLHF score	−15.79 ± 10.77	<0.001	−10.76 ± 9.94	<0.001	−5.30 (−8.33, −2.28)	<0.001

*Values are presented as mean ± SD*.

* *Delta is the difference between 6-month measurements and the baseline value*.

# *P-value is for the comparison within groups*.

## *P-value was used for comparison between groups*.

† *Multivariable regression models were used to explore the difference of outcomes between two groups when adjusted for clinical covariates, QLV/QRS*.

‡ *The effect size is the adjusted difference of “Delta” for index between two groups (BiV+SyncAV group vs. BiV group). The adjusted difference was calculated based on the multivariable regression models*.

### Echocardiographic Responses

**Figure 3:**
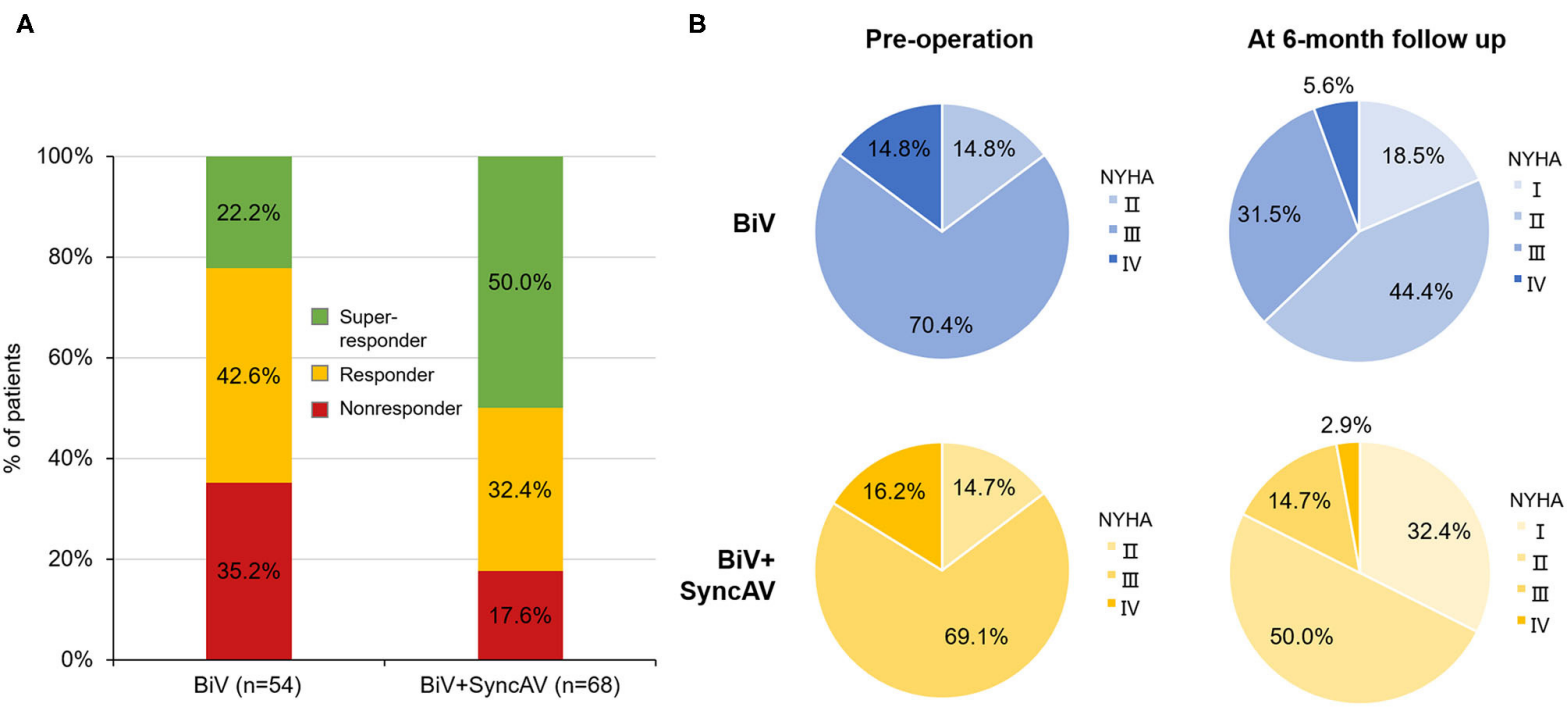
**(A)** Proportion of non-responder, responder, and super-responder in the BiV+SyncAV group and BiV group; **(B)** New York Heart Association functional class (NYHA) at baseline and 6-month follow-up between BiV+SyncAV group and BiV group. BiV, biventricular pacing.

The BiV and LV only+SyncAV (50 ms offset) modes produced higher aVTI scores than intrinsic (BiV vs. intrinsic: 22.34 ± 4.65 cm vs. 19.58 ± 4.44 cm, *P* < 0.001; LV only+SyncAV vs. intrinsic: 22.02 ± 4.58 cm vs. 19.58 ± 4.44 cm, *P* < 0.01), whereas, the highest aVTI was acquired in the BiV+SyncAV modes (23.96 ± 4.65 cm, *P* < 0.001) (Figure 2B). After 6-month follow-up, the ultrasonic results of the two groups were significantly better than those before operation. Compared to BiV settings, BiV+SyncAV resulted in more significant decreases in LVESV (−28.86 ml, 95% CI −38.09 ~ −19.64 ml; *P* < 0.001) and larger increases in LVEF (7.82%, 95% CI 5.53 ~ 10.11 %; *P* < 0.001) (Table 2 and Supplementary Table 4). More echocardiographic responders were observed in the BiV+SyncAV group than in the BiV group (56 [82.35%] vs. 35 [64.81%]; *P* = 0.036; Figure 3A). The rate of superresponders in the BiV+SyncAV group was significantly higher than that in the BiV group (34 [50.00%] vs. 12 [22.22%]; *P* = 0.002; Figure 3A). There was no significant difference in LVESV at 6 months between patients with QLV/QRS >0.7 and QLV/QRS ≤0.7 in the BiV+SyncAV group (−69.97 ± 38.98 vs. −32.96 ± 27.20 ml, *P* = 0.115, Supplementary Table 2).

### Clinical Responses

During follow-up, the optimization of the BiV+SyncAV improved the clinical response compared to the BiV mode in terms of the 6MWT, MLHF score, and NYHA classification (Figure 3B). At the end of the 6-month follow-up period, BiV+SyncAV resulted in a statistically significant difference in the decrease in the MLHF score (−5.30, 95% CI −8.33 ~ −2.28; *P* < 0.001) and an increase in the 6MWT distance (67.18 m, 95% CI 49.93 ~ 84.44 m; *P* < 0.001) compared with BiV (Table 2). Clinical response, defined as a drop of ≥1 NYHA class relative to baseline, was seen in 57 patients (83.82%) in the BiV+SyncAV group compared to 36 in the BiV group (66.67%, *P* = 0.033).

### Adverse Events

During follow-up, no significant differences in the medication condition and pacemaker-related complications were found between groups. In the BiV+SyncAV group, a single case of LV lead dislocation occurred in the BiV+SyncAV group after CRT implantation, so it was repositioned. In the BiV group, a single case of infection was observed following pacemaker implantation and required removal and reimplantation of the CRT device on the opposite side after 1 month. Five (7.35%) BiV+SyncAV patients and 7 (12.96%) BiV patients required rehospitalization for HF deterioration. In total, 8/122 patients (5 in the BiV+SyncAV group; 3 in the BiV group) showed phrenic nerve stimulation at single pacing sites that resolved following the reprogramming of the pacing sites, reduction of left ventricular output, or lengthening of the pacing pulse width, eventually ensuring effective left ventricular pacing. No deaths due to HF deterioration or early pacemaker battery depletion occurred during follow-up.

## Discussion

To the best of our knowledge, this is the first comparison of the echocardiographic response and clinical improvement by CRT with vs. without SyncAV-based AVD. We found that (1) BiV+SyncAV modes led to higher aVTI scores than BiV, indicating improved acute hemodynamics; (2) SyncAV performance not only enhanced electrical parameters but also improved echocardiographic and clinical responses; (3) subgroup analysis revealed that patients with QLV/QRS >0.7 displayed comparable QRSd and echocardiographic parameters to patients with QLV/QRS ≤0.7, suggesting that the location of the LV lead does not influence the efficacy of SyncAV.

Traditional BiV pacing is non-physiological due to retrograde excitation of the ventricular septum, limiting the benefits of BiV pacing, which can be improved through BiV+intrinsic fusion. Horst and colleagues demonstrated that the maximum rates of LV pressure increase (LV dP/dtmax) occur when BiV is fused with pacing and intrinsic conduction (9). Guo et al. (22) showed that BiV+intrinsic pacing following ECG optimization in CHF patients improved cardiac function and quality of life. The optimal AVD therefore achieves the complete fusion of BiV pacing and intrinsic AV conduction. AVD is also challenging due to post-implant conditions in response to exercise, heart rate, autonomic tone, or drugs. Clinical optimization therefore fails to accommodate the dynamics of individual patients (23).

Truly effective CRT optimization comes from individualized programming, as opposed to “out-of-the-box” settings. SyncAV can lead to dynamic fusion of BiV pacing and intrinsic AV conduction, reduce QRS duration significantly, and eventually produce the best electrical resynchronization. Consistent with previous studies (10, 11), our data showed that BiV pacing with individualized SyncAV offsets yielded the narrowest QRS. Thibault et al. (11) also showed that either BiV_EarlyLV_ or BiV_LateLV_ electrode resulted in the same shortened QRSd using SyncAV optimization at the default 50-ms offset. Similar results were obtained in our subgroup analysis that showed that QLV/QRS ratio did not influence the efficacy of SyncAV. Overall, the SyncAV algorithm has a broad range of application prospects in clinical practice and may benefit HF patients with intact AV conduction.

Other algorithms to automatic AVD optimization include Adaptiv-CRT (Medtronic) that provides LV-only pacing (AVD ≤200 ms) and BiV pacing (AVD >200 ms) (24). The SyncAV and Adaptiv-CRT algorithms have some common and individual characteristics. They all depend on the sinus rhythm and intact atrioventricular conduction function and encourage intrinsic right ventricular activation. Birnie et al. (25) indicated that Adaptiv-CRT resulted in the improvement of clinical outcomes, and the benefit of Adaptiv-CRT for the improvement of cardiac function was mainly derived from LV-only pacing. SyncAV permits wider AVD (≤325 ms) programming using the “optimal” offset (Supplementary Figure 4), which results in triple wavefront fusion (right bundle branch conduction, RV paced activation, and LV paced activation). The SyncAV and Adaptiv-CRT algorithms have their own characteristics, and further, research is needed to determine their own suitable population.

Post-implant electrical optimization achieves acute hemodynamics or long-term LV remodeling as the ultimate therapeutic goal (26). The gold standard for evaluating acute hemodynamic performance in CRT is to measure the rate of LV pressure rise, which is invasive, time-consuming, and costly. In contrast, measurement of aVTI is relatively simple and non-invasive. There is evidence that aVTI is a reliable indicator of cardiac output during CRT optimization (27, 28). Sometimes it is challenging to distinguish the difference in QRSd milliseconds between different offsets, and aVTI can be used as a valuable auxiliary indicator to distinguish such subtle differences. In our study, compared with BiV mode or LV only+SyncAV 50-ms mode, the BiV+SyncAV modes yielded the highest aVTI scores. Similarly, Wang et al. (29) also demonstrated that shortening of QRSd is closely related to the acute improvement of hemodynamic parameters.

Of note, the 6-month follow-up performed in this study showed that the SyncAV improved LV remodeling, as evidenced by the lower LVESV and higher LVEF than in BiV mode. The reported incidence of echocardiographic response after CRT implantation in the literature ranges between 60 and 75% (30). Consistent with previous results, our data showed that 82.35% patients in the BiV+SyncAV group vs. 64.81% in the BiV group were classified as echocardiographic responders (*P* = 0.036). After 6 months of follow-up, super-responses were significantly more common in the BiV+SyncAV group than that in the BiV group (50.00 vs. 22.2%, *P* = 0.002). Previous study demonstrated that the early response to CRT treatment predicts a favorable prognosis (31). Accordingly, we conclude that SyncAV dynamically optimizes the AV interval to achieve triple wavefront fusion and eventually contributes to improving clinical results of CRT patients. As predicted, NYHA cardiac functional grading, 6-min walking distance, and MLHF significantly improved in the BiV+SyncAV group compared with the BiV group 6 months after implantation.

### Study Limitations

First, our study is a retrospective study, but it is the first time to demonstrate the SyncAV function on echocardiographic and clinical improvements in CRT patients. We used multivariate regression models to adjust some parameters potentially affecting the improvement of cardiac function. We also conducted analyses with two propensity score (IPTW and OW) weighting methods to reduce the effects of confounders. After weighting, the baseline characteristics between two group had been well-balanced (Supplementary Figures 2, 3). The results of sensitivity analyses showed that the QRS, clinical, and echocardiographic outcomes were consistent with those shown in Supplementary Table 3. Second, VV interval was fixed at 30 ms in our study instead of optimal VV interval. While optimizing the SyncAV offset value, it is worth exploring whether to optimize the VV interval and the best timing of the VV interval. Third, the majority of enrolled subjects had dilated heart disease. It thus remains unclear whether patients with ischemic cardiomyopathy with heavy LV scarring can benefit from SyncAV. Finally, the number of patients was small, so further, RCTs with larger sample sizes and longer follow-up periods are still needed to confirm our findings.

## Conclusion

This multicenter observational cohort trial revealed that BiV pacing coupled to the SyncAV function can narrow the QRSd in CRT patients and a reversal of LV remodeling compared to conventional BiV pacing. Cardiac function benefits from the dynamic fusion of instinct conduction and BiV pacing. SyncAV therefore should be applied in all CRT patients with normal instinct conduction.

## Data Availability Statement

The raw data supporting the conclusions of this article will be made available by the authors, without undue reservation.

## Ethics Statement

The studies involving human participants were reviewed and approved by Changhai Hospital, Second Military Medical University. The patients/participants provided their written informed consent to participate in this study.

## Author Contributions

ZW: collection and analysis. PL: data collection and writing. BZ: data interpretation and analysis. JH: literature search. SC: manuscript preparation. ZC: figures preparation. YiQ: statistical analysis. JF: tables preparation. WT: data interpretation. YoQ: literature search. RL: data interpretation and literature search. XZ: study design and writing the manuscript. All authors contributed to the article and approved the submitted version.

## Conflict of Interest

The authors declare that the research was conducted in the absence of any commercial or financial relationships that could be construed as a potential conflict of interest.

## Publisher's Note

All claims expressed in this article are solely those of the authors and do not necessarily represent those of their affiliated organizations, or those of the publisher, the editors and the reviewers. Any product that may be evaluated in this article, or claim that may be made by its manufacturer, is not guaranteed or endorsed by the publisher.
