# Supplementary material for: Improvement of LV Reverse Remodeling Using Dynamic Programming of Fusion-Optimized Atrioventricular Intervals in Cardiac Resynchronization Therapy
Source: Front Cardiovasc Med. 2021 Aug 20;8:700424. doi: 10.3389/fcvm.2021.700424 (PMC8417774; doi:10.3389/fcvm.2021.700424)

**SupplementalTable 1** Electrode characteristics

| Characteristics | **Overall**  **(n =122)** | **BiV+SyncAV group**  **(n =68)** | **BiV group**  **(n =54)** | ***P*-Value** |
| --- | --- | --- | --- | --- |
| Circumferential position, n (%)  Anterior(n, %)  Anterolateral(n, %)  Lateral(n, %)  Posterlateral(n, %)  Posterior(n, %) | 6 (4.9)  21 (17.2)  58 (47.5)  24 (19.7)  13 (10.7) | 3 (4.4)  12 (17.6)  32 (47.1)  13 (19.1)  8 (11.8) | 3 (5.6)  9 (16.7)  26 (48.1)  11 (20.4)  5 (9.2) | 0.871 |
| Longitudinal position, n (%)  Mid (n, %)  Base (n, %)  Apex (n, %) | 72 (59.0)  34 (27.9)  16 (13.1) | 40 (58.8)  19 (27.9)  9 (13.3) | 32 (59.3)  15 (27.8)  7 (12.9) | 0.957 |
| QLV (ms) | 110.0 (100.0, 125.0) | 110.0 (100.0, 125.0) | 115.0 (100.0, 128.0) | 0.362 |
| QLV/QRSd | 0.70±9.9 | 0.70±10.6 | 0.70±8.9 | 0.974 |
| RV-LV interval (ms) | 78.0 (70.0, 89.0) | 76.0 (70.0, 88.0) | 79.0 (70.0, 90.0) | 0.509 |

## Values are presented as mean±SD, median(IQR) or n (%) as appropriate.

**Supplemental Table 2**Subgroup analyses of QLV/QRSd (BiV+SyncAV group).

| Outcomes | QLV/QRSd ≤ 0.7  (N=29) | QLV/QRSd >0.7  (N=39) | Multivariable analyses^#^ | |
| --- | --- | --- | --- | --- |
|  |  |  | Effect Size (95% CI)**^‡^** | P-value^*^ |
| Delta QRS,ms | -33.59±14.52 | -38.23±17.47 | -5.70 (-17.74, 6.35) | 0.344 |
| Delta LVEF, % | 8.47±6.63 | 17.41±8.56 | 4.33 (-1.88, 10.53) | 0.166 |
| Delta LVEDV, ml | -40.93±36.60 | -75.35±46.54 | -20.34 (-51.24, 10.56) | 0.190 |
| Delta LVESV, ml | -32.96±27.20 | -69.97±38.98 | -18.84 (-42.46, 4.79) | 0.115 |

Values are presented as mean ± SD.

^*^P value was used for comparison between groups.

^#^Multivariable regression models were used to explore the difference of outcomes between two groups when adjusted for clinical covariates,QLV/QRS.

^‡^The effect size is the adjusted difference ofDeltafor index between two groups (BiV+SyncAV group vs. BiV group). The adjusted difference was calculated based on the multivariable regression models.

**SupplementalTable 3**Propensity Score Analyses for Improvement in QRS, Clinical, and Echocardiographic Outcomes Between Groups.

|  | IPTW | | OW | |
| --- | --- | --- | --- | --- |
| Outcomes | Effect Size (95% CI) | P-value | Effect Size (95% CI) | P-value |
| Delta QRS, ms | -11.14 (-17.63, -4.64) | 0.001 | -11.51 (-17.91, -5.12) | 0.001 |
| Delta LVEF, % | 7.51 (4.26, 10.76) | <0.001 | 7.78 (4.53, 11.03) | <0.001 |
| Delta LVESV, ml | -27.75 (-41.10, -14.40) | <0.001 | -28.33 (-41.75, -14.91) | <0.001 |
| Delta LVEDV, ml | -35.02 (-50.40, -19.64) | <0.001 | -36.37 (-51.72, -21.01) | <0.001 |
| Delta 6MWT, m | 64.25 (44.21, 84.29) | <0.001 | 65.67 (45.58, 85.77) | <0.001 |
| DeltaMLHF score | -4.84 (-8.54, -1.13) | 0.011 | -5.17 (-8.89, -1.44) | 0.007 |

IPTW: Shown is the effect size from the univariate analyses with inverse probability weighting according to the propensity score.

OW: Shown is the effect size from the univariate analyses with overlap weighting according to the propensity score.

**Supplement Table 4** Comparison of LVESV/BSA and LVEDV/BSA before and after CRT implantation.

| Characteristics | Overall  (n =122) | BiV+SyncAV  (n =68) | BiV  (n = 54) | *P*-Value |
| --- | --- | --- | --- | --- |
| LVEDV/BSA basal (ml/m^2^) | 117.3 (94.1, 139.6) | 115.1 (94.0, 137.9) | 118.6 (94.8, 141.9) | 0.887 |
| LVESV/BSA basal (ml/m^2^) | 75.9 (61.0, 93.8) | 74.5 (61.4, 93.4) | 77.3 (59.5, 93.8) | 0.893 |
| LVEDV/BSA post CRT (ml/m^2^) | 89.3 (69.4, 117.8) | 77.1 (65.7, 103.0) | 99.6 (79.1, 127.4) | 0.002 |
| LVESV/BSA post CRT (ml/m^2^) | 51.4 (35.4, 77.3) | 44.0 (31.1, 59.2) | 61.2 (42.1, 82.4) | 0.002 |

**Supplemental Figure 1**

The position of the LV electrode is usually determined by two project positions. Under the 45° left anterior oblique, the left ventricle can be divided into anterior, anterolateral, lateral, posterolateral and posterior segments. Under the 30° right anterior oblique, the left ventricle can be divided into base, middle and apex segments.

**
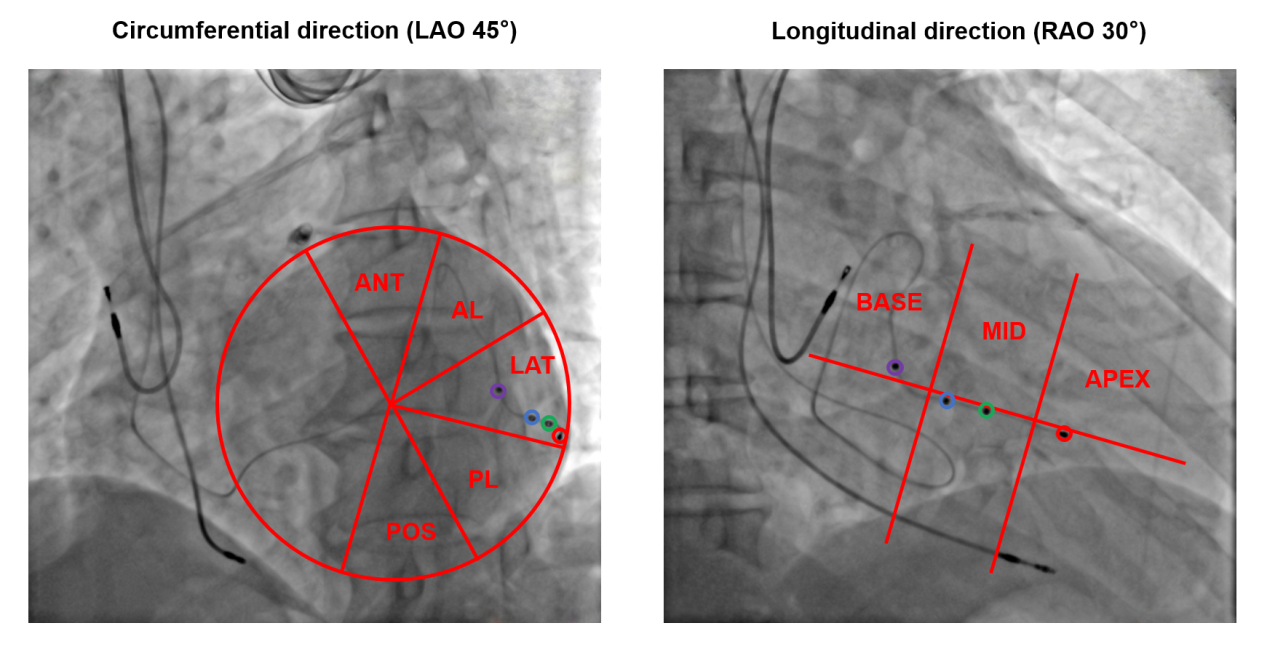
**

**Supplemental Figure 2** The absolute standardized mean differences to evaluate the balance of covariates between two groups basedoninverseprobabilityweighting.


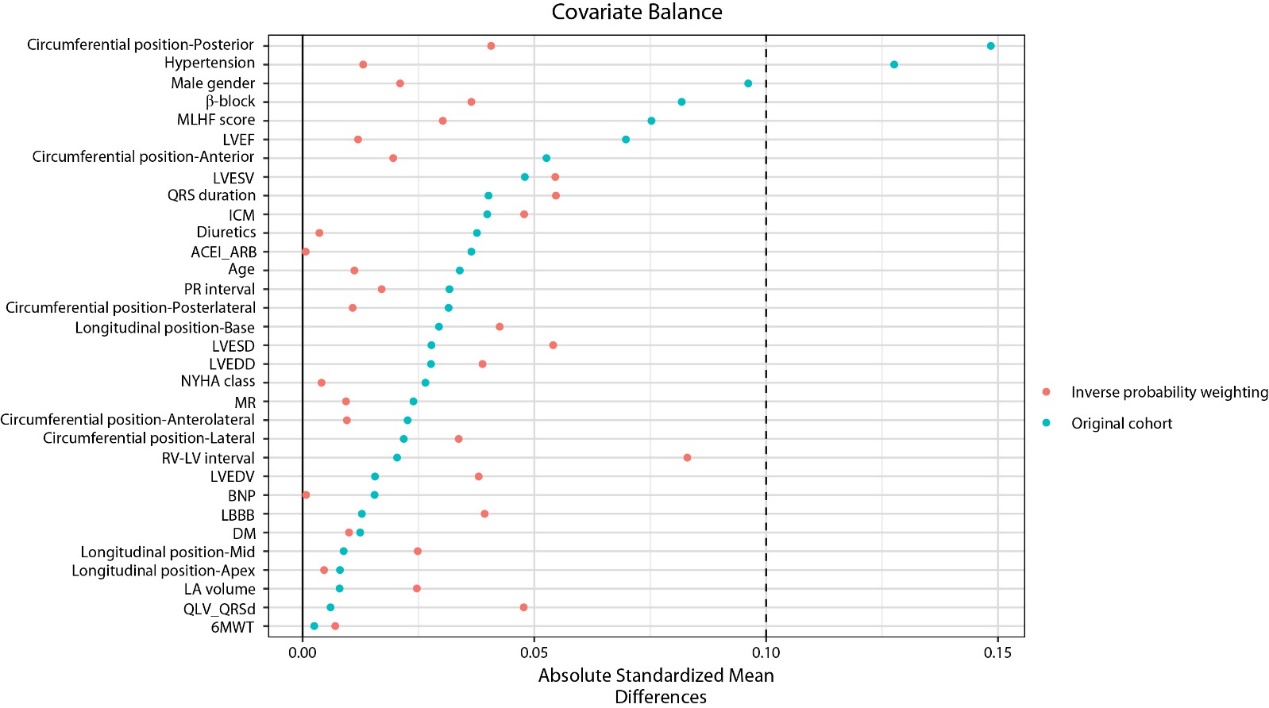


**Supplemental Figure 3** The absolute standardized mean differences to evaluate the balance of covariates between two groups basedon overlap weighting.


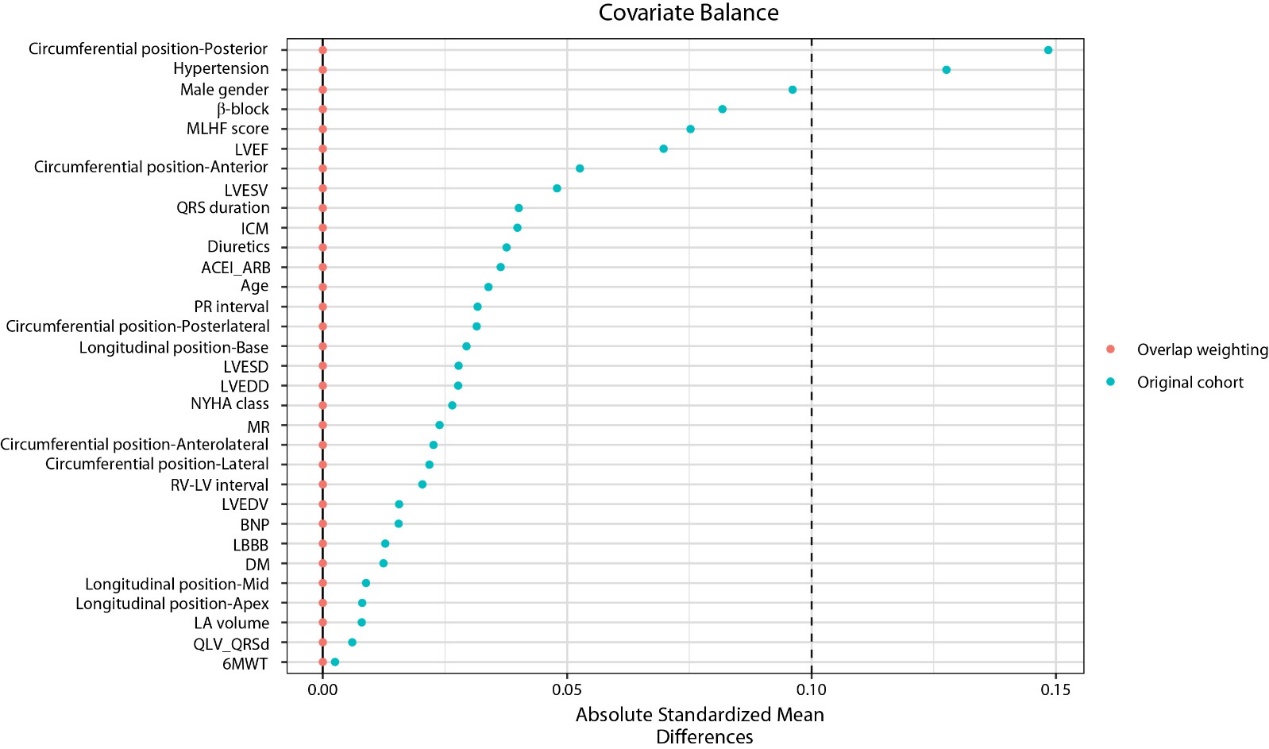


**Supplemental Figure 4**Distribution of optimal SyncAV offsets.


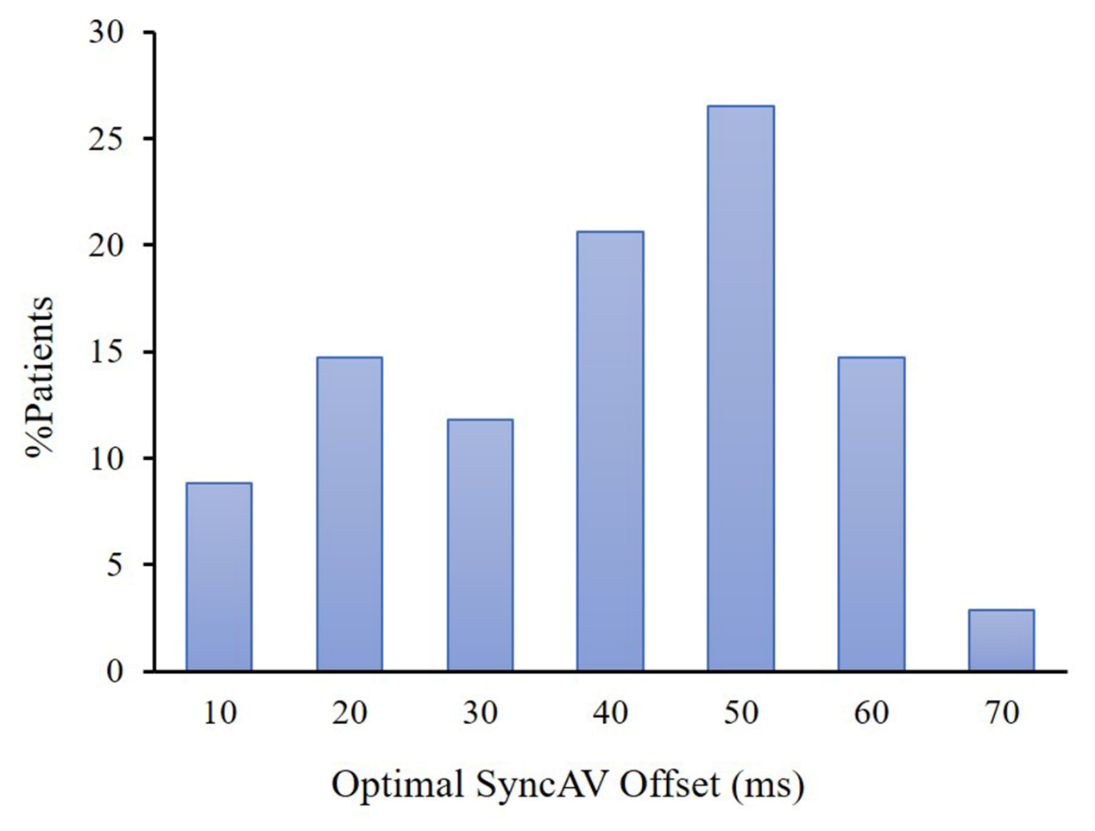

Supplement: Supplementary file 1 [file Data_Sheet_1.docx]
